# Exploration of laser-driven electron-multirescattering dynamics in high-order harmonic generation

**DOI:** 10.1038/srep32763

**Published:** 2016-09-06

**Authors:** Peng-Cheng Li, Yae-Lin Sheu, Hossein Z. Jooya, Xiao-Xin Zhou, Shih-I Chu

**Affiliations:** 1 College of Physics and Electronic Engineering, Northwest Normal University, Lanzhou 730070, China; 2 Center for Quantum Science and Engineering, and Center for Advanced Study in Theoretical Sciences, Department of Physics, National Taiwan University, Taipei 10617, Taiwan; 3 Department of Chemistry, University of Kansas, Lawrence, Kansas 66045, USA

## Abstract

Multiple rescattering processes play an important role in high-order harmonic generation (HHG) in an intense laser field. However, the underlying multi-rescattering dynamics are still largely unexplored. Here we investigate the dynamical origin of multiple rescattering processes in HHG associated with the odd and even number of returning times of the electron to the parent ion. We perform fully *ab initio* quantum calculations and extend the empirical mode decomposition method to extract the individual multiple scattering contributions in HHG. We find that the tunneling ionization regime is responsible for the odd number times of rescattering and the corresponding short trajectories are dominant. On the other hand, the multiphoton ionization regime is responsible for the even number times of rescattering and the corresponding long trajectories are dominant. Moreover, we discover that the multiphoton- and tunneling-ionization regimes in multiple rescattering processes occur alternatively. Our results uncover the dynamical origin of multiple rescattering processes in HHG for the first time. It also provides new insight regarding the control of the multiple rescattering processes for the optimal generation of ultrabroad band supercontinuum spectra and the production of single ultrashort attosecond laser pulse.

In the past twenty years, the process of laser-atom interactions in intense laser fields has attracted much attention due to the highly nonlinear optical phenomena^1^,^2^,^3^, such as multiphoton ionization, above-threshold ionization, and high-order harmonic generation (HHG), etc. In particular, HHG provides a promising method to produce the coherent extreme ultraviolet (XUV) light source in the attosecond time scale^4^,^5^,^6^, leading to novel applications such the observation and control of the real-time electronic dynamical behavior^7^,^8^,^9^. The laser-atom interactions in HHG can be understood by the semiclassical three-step model^10^,^11^. Within this physical model, an electron firstly tunnels through the barrier formed by the coulomb potential and the laser field, and then it is accelerated by an applied laser field. Finally, the electron can be driven back toward the parent ion to recombine to the ground state and emits the harmonic photons. The HHG spectra typically have the approximately constant amplitude of a broad spectral range called the plateau. The plateau is followed by a sharp cutoff beyond which no further harmonic emission is seen. The major attention of the HHG study has been focused on how to enhance the HHG plateau and extend the harmonic cutoffs for the generation of bright coherent XUV source.

Recently, Popmintchev *et al*.^12^ have shown experimentally that the enhanced phase-matched harmonic generation can be generated by means of mid-infrared laser fields, allowing the significant extension of the cutoff of HHG to keV energies. The process of multiple rescattering is expected to play an important role in the laser-atom interactions driven by the midinfrared laser fields^13^,^14^,^15^, but the dynamical origin of multiple rescattering processes in HHG of atoms is still less understood and largely unexplored. Miller *et al*.^16^ have theoretically studied the control of the emergence of multiple wave-packet rescattering in the process of HHG by using an isolated attosecond vacuum ultraviolet pulse. Tong *et al*.^17^ have presented a quantitative study of the low energy structure (LES) of strong field ionization by mid-IR laser pulses and found that the photoelectron angular distributions are due to multiple rescattering of the electron in the laser field. More recently, we have shown^18^ that the laser-driven electron-multiple rescattering have strong contributions in resonance-enhanced below-threshold harmonic generation of the atoms.

In this paper, we present a new finding of the dynamical origin of multiple rescattering processes in HHG of atoms associated with odd and even number of returning times of the electron to the parent ion. The harmonic spectrum of hydrogen atom can be calculated accurately and efficiently by solving the time-dependent Schrödinger equation (TDSE) by means of the time-dependent generalized pseudospectral method (TDGPS)^19^. The TDGPS method allows for *non-uniform* and optimal discretization of the spatial coordinates and it has shown to be capable of providing accurate results for both the bound-state as well as the time-dependent wave functions and HHG rates (from the lowest to the cutoff harmonics), etc^19^. As another example, in ref. 20, it is shown that the eigen-energies of various H_2_^+^ electronic states can be determined up to 28 digits of accuracy with the use of only a minimal number of spatial grid points.

To explore the dynamical origin of the multiple rescattering processes, we extend an empirical mode decomposition (EMD) method^21^ to extract the multiple rescattering contributions in HHG and present the corresponding time-frequency representations using synchrosqueezing transform (SST)^22^,^23^. In addition, we apply the standard semiclassical approach suggested independently by Corkum^10^ and Kulander *et al*.^11^ to obtain the electron trajectories. By comparing the quantitative time-frequency representations and the extended semiclassical calculations, we are able to distinguish the contributions of the short trajectories and long trajectories in multiple rescattering processes in HHG of atoms. We find that the short trajectories with odd number of rescattering times are dominant when the tunneling ionization regime is involved, while the long trajectories with even number of rescattering times are dominant when the multiphoton ionization regime is involved. The multiphoton- and tunneling-ionization regimes in multiple rescattering processes occur alternately. This new finding of the dynamical origin of multiple rescattering processes can provide fresh new insight towards the optimal control of the multiple rescattering processes to facilitate the generation of ultrabroad band supercontinuum spectra as to be discussed in a later section.

## Laser-Driven Electron-Multirescattering

**Figure 1:**
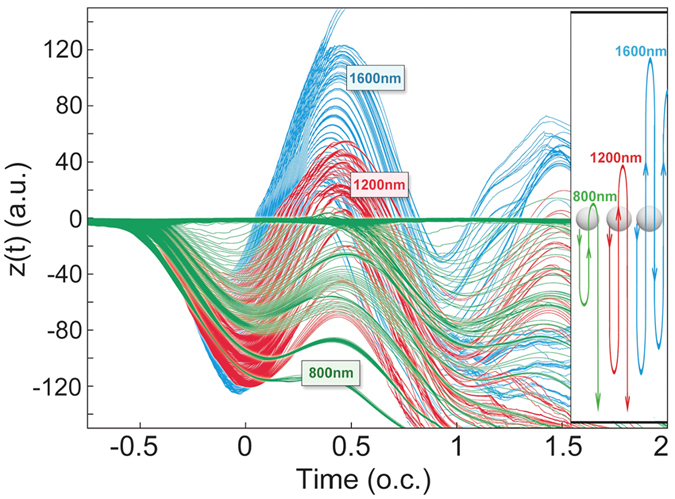
Multiple rescattering trajectories calculated by Bohmian mechanics. In the Bohmian mechanics frame, multiple rescattering trajectories are given as position versus the time of hydrogen atom driven by an intense 800-nm (green lines), 1200-nm (red lines), and 1600-nm (blue lines) laser pulse with the peak intensity *I* = 2.0 × 10^14^ W/cm^2^ at −0.1 optical cycle.

To understand the physical picture of multiple rescattering processes in HHG, Fig. 1 shows the Bohmian trajectories^24^ of hydrogen atom driven by an intense 800-nm, 1200-nm, and 1600-nm laser pulse with the peak intensity *I* = 2.0 × 10^14^ W/cm^2^ at −0.1 optical cycle, respectively. The corresponding Keldysh parameter 
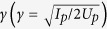
 is equal to 1.1, 0.71, and 0.53 for the 800-nm, 1200-nm, and 1600-nm laser pulse, respectively. Note that the multiphoton ionization is typically classified by *γ* > 1, while tunneling ionization is by *γ* < 1. We adopt a laser pulse with the cosine-squared shape and the duration of 10 optical cycles. The Bohmian trajectories of the hydrogen atom can be calculated by Bohmian mechanics. The theoretical framework and the numerical approach are presented in Methods section.

In the de Broglie-Bohm framework, the individual tracer particles are evolved along quantum trajectories with the velocities generated by the time-dependent wave function field. The patterns developed by these quantum trajectories as they emerge from an ensemble of initial points exactly define the history of the system as it evolves from the initial to final state. This allows one to employ de Broglie–Bohm’s framework of Bohmian mechanics (BM) to provide an accurate trajectory-based scheme to interpret the electron wave packet dynamics^25^. Strong-field approximation (SFA) is often used to provide qualitative trajectory based insight about the electron dynamics in intense laser-atom interactions. An extended SFA is recently developed to explore the conditions for resolving high-order electronic re-collisions in high-order harmonic spectroscopy^26^,^27^. Within the SFA framework, it has been shown that the inclusion of the electron-core Coulomb interaction is inevitable in intense field atomic processes^28^,^29^,^30^. In our present fully *ab initio* approach, we go beyond SFA and exact Coulomb potential is used in our calculation without any approximation.

The inset illustrates the multiple rescattering processes for different the laser wavelength. As discussion above, the De Broglie-Bohm’s framework of Bohmian mechanics provide an accurate trajectory-based scheme to causally interpret the electron wave packet dynamics. According to the Bohmian trajectories, the multiple rescattering processes is evident in HHG, particularly in the long-wavelength laser field (mid-infrared). Therefore, as an example, we adopt an 1600-nm laser pulse to explore the dynamical origin of multiple rescattering processes in HHG which associates with odd and even number of returning times of the electron to the parent ion.

## High-order Harmonic Spectra and Semiclassical Trajectories

**Figure 2:**
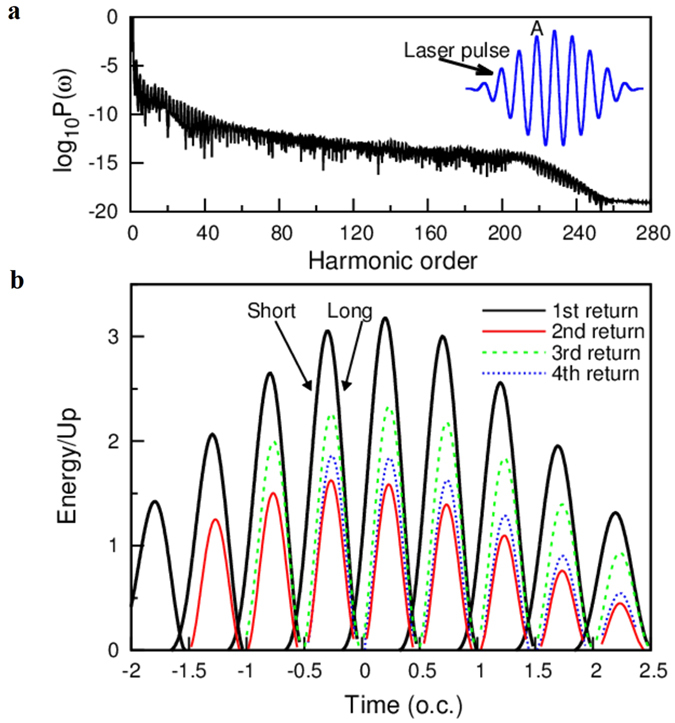
HHG spectra, and semiclassical trajectories including the multiple rescattering processes. (**a**) The HHG power spectrum of hydrogen driven by an intense 1600-nm laser pulse with the peak intensity *I* = 2.0 × 10^14^ W/cm^2^. The blue solid line indicates the laser pulse. The other laser parameters used are the same as those in Fig. 1. (**b**) Semiclassical returning energy as a function of the returning time. The black arrows indicate the short trajectory (Short) and long trajectory (Long) at the peak of the ionization time marked by “A” in Fig. 2a.

In Fig. 2a, we present the HHG power spectrum of atomic hydrogen driven by an intense 1600-nm (mid-infrared) laser pulse with the peak intensity*I* = 2.0 × 10^14^ W/cm^2^. The other laser parameters used are the same as those in Fig. 1. In Fig. 2b, the semiclassical returning energy as a function of returning time is shown. The maximum returning energy located at around 3.2*U*_*p*_ corresponds to the HHG cutoff (about the 210^th^ harmonic order), this semiclassical calculation is in good agreement with the HHG power spectrum shown in Fig. 2a. In addition, the maximum return energy of the second rescattering is about 1.5*U*_*p*_ (about the 95^th^ harmonic order), the maximum return energy of the third rescattering processes is about 2.0*U*_*p*_ (about the 125^th^ harmonic order). Although the semiclassical simulation can qualitatively provide the maximum return energy of the first rescattering and multiple rescattering, the quantitative contributions of the short- and long-trajectories in multiple rescattering processes are not revealed. Therefore, we adopt an extended semiclassical method^31^ to calculate the probability of the electrons with the corresponding returning time *t* and returning energy*E*, which can be obtained from the following expression:


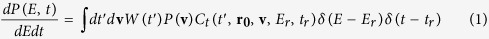


where *W*(*t*′) is the instantaneous ionization rate which can be defined as a logarithmic derivative of the time-dependent population *P*(*t*′) calculated by solving TDSE^32^, i.e., 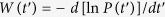, *t*_*r*_ and *E*_*r*_ are the returning time and returning energy for given trajectories, respectively, and *P*(**v**) is the Gaussian initial velocity distribution. The instantaneous ionization rate *W*(*t*′) is a function as the time *t*′ of the laser field. Each time *t*′ has an instantaneous ionization rate *W*(*t*′), but we only need to consider the instantaneous ionization rate of the electrons with the corresponding return time *t*_*r*_ and the return energy *E*_*r*_. Note that the electron cannot return to the parent ion at the time *t*′ of the laser field in some cases, *t*_*r*_ and *E*_*r*_ depends on the initial position **r**_**0**_ and the initial velocity **v** of the electron. So, in calculation, each trajectory is monitored for all the approaches to the parent ion. If an electron trajectory is such that it can return to the parent ionic core at time *t*_*r*_ with returning kinetic energy *E*_*r*_, the factor *C*_*t*_(*t*′, **r**_**0**_, **v**, *E*_*r*_, *t*_*r*_) is set to 1. Otherwise, *C*_*t*_(*t*′, **r**_**0**_, **v**, *E*_*r*_, *t*_*r*_) = 0.

**Figure 3:**
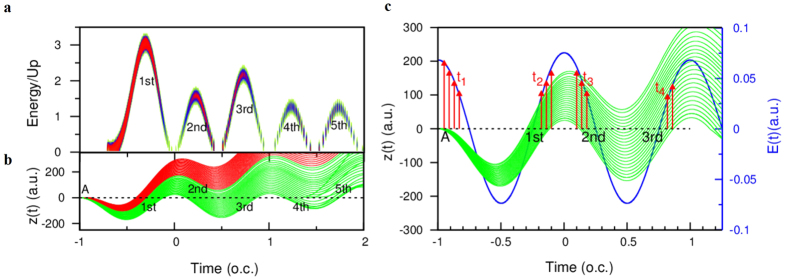
Return energy map & semiclassical trajectories with the multiple rescattering processes. (**a**) The probability of the electrons with the corresponding returning time *t* and returning energy *E* calculated by an extended semiclassical method. Note that the semiclassical calculation corresponds to the peak of the ionization time marked by “A” in Fig. 2a. (**b**) Several short trajectories (red lines) and long trajectories (green lines) in HHG and the corresponding first returning and multiple returning are marked by the 1^st^, 2^nd^, 3^rd^, 4^th^, and 5^th^, respectively. The laser parameters used are the same as those in Fig. 2a. (**c**) Time-dependent laser fields (blue line) and semiclassical long trajectories, the red arrows indicate the peak intensity of laser field with the corresponding ionization time of first returning and multiple returning. Note that every electron trajectory of the first returning starts at the peak A and time t_1_, every electron trajectory of the multiple returning restarts at time t_2_, t_3_, and t_4_, respectively.

In Fig. 3a, the probability of the electrons with the corresponding return time *t* and return energy *E* is presented. Note that the semiclassical calculation corresponds to the ionization time marked by the peak A in Fig. 2a. We find that the first returning (1^st^) and all the multiple returnings (2^nd^, 3^rd^, 4^th^ and 5^th^) have two emission bursts resulting harmonic generation and occur at each half optical cycle. Figure 3b shows several short trajectories and long trajectories in HHG. It is clear that the short trajectories have no multiple returns, only the long-trajectories electrons show the multiple rescatterings. The corresponding first returning and multiple returning are marked by 1^st^, 2^nd^, 3^rd^, 4^th^ and 5^th^, respectively. Although the multiple returns only belong to the long trajectories, as the result shown in Fig. 3a, the multiple returns still show two emission bursts at each half optical cycle. Namely, each return (the first returning and multiple returning) have two dominant quantum trajectories that contribute to each harmonic emission in the half optical cycle. So we still can call these dominant quantum trajectories associated with multiple returning as the short- and long-trajectories at each half optical cycle. Therefore, it is clear that the short trajectories in the 1^st^, 3^rd^ and 5^th^ returning (odd number times rescattering) are dominant, while the long trajectories of the 2^nd^ and 4^th^ returning (even number times rescattering) are dominant. In Fig. 3c, the time-dependent position of electrons and the corresponding time-dependent laser fields are presented, the red arrows indicate the peak intensity of laser field with the corresponding ionization time of the first returning and multiple returning. Note that every electron trajectory of the first returning starts at the peak A and at time t_1_, and every electron trajectory of the multiple returning restarts at time t_2_, t_3_, and t_4_, respectively.

For the 1^st^ returning electrons, the region of the corresponding ionization time (t_1_) is located at the falling edge of near the peak of laser pulse, the electrons tunnel through the barrier and return the parent ion when the laser field changes the sign. However, the electrons of the 2^nd^ returning are ionized at the rising edge of laser pulse (t_2_), the sign of the laser field is impossible to be changed in the half optical cycle, so only the electrons are pulled back quickly to the core due to the movement of the electrons against the force of the laser field. According to our previous studies^15^, the multiphoton ionization is dominant in this kind of process. For the 3^rd^ returning electrons, the region of the corresponding ionization time (t_3_) is located at the falling edge of the laser pulse, therefore this regime is similar to the 1^st^ returning electrons, i.e., the tunneling regime is dominant, only that the return energy is smaller. The reason is that the laser intensity with the corresponding ionization time is smaller than those of the 1^st^ returning electrons. In addition, the ionization regime of the 4^th^ returning electrons is similar to the 2^nd^ returning electrons. Therefore, we find that the multiphoton- and tunneling-ionization regimes in odd- and even-number times of rescattering processes occur alternatively.

## Electron Dynamics in the Multiple Rescattering Processes

**Figure 4:**
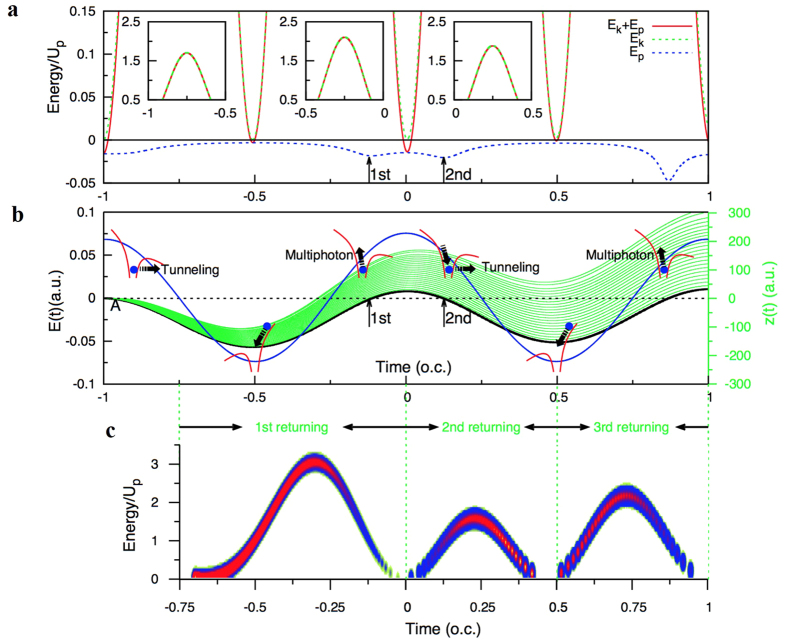
Scheme of electron dynamics and instantaneous energy associated with semiclassical trajectories. (**a**) The total instantaneous energy, kinetic energy *E*_*k*_, and potential energy *E*_*P*_ of a given long-trajectory electron. The insets indicate the total instantaneous energy, kinetic energy *E*_*k*_ from 0.5 a.u. to 2.5 a.u as a function of the time. The black arrows indicate the time of the 1^st^ returning and the 2^nd^ returning. (**b**) Time-dependent laser fields *E*(*t*′) (blue line) and scheme of the electron dynamics are illustrated. The green lines indicate the corresponding semiclassical long trajectories. The insets (bottom) labeled by **c** indicate the return energies associated with the 1^st^ returning, the 2^nd^ returning, and the 3^rd^ returning, respectively. The color map of the return energy plane indicates the dominant contributions to the trajectories.

To understand the dynamical origin of multiple rescattering processes in HHG of atoms which associate with the odd and even number times of returning of the electron to the parent ion, we present the total instantaneous energy, kinetic energy *E*_*k*_, and potential energy *E*_*P*_ of a given long-trajectory electron (black solid line shown in Fig. 4b) in Fig. 4a. The insets indicate the total instantaneous energy, kinetic energy *E*_*k*_ from 0.5 a.u. to 2.5 a.u as a function of the time. The black arrows indicate the time of the 1^st^ returning and the 2^nd^ returning. In Fig. 4a, the potential energy *E*_*P*_ of a given long-trajectory electron is increasing after the 1^st^ returning, and the total instantaneous energy (*E*_*k*_ + *E*_*P*_) is negative before the second returning. This result implies that the 1^st^ returning electrons recombine to parent ion followed by the absorption of many photons and quickly return to the core (the 2^nd^ returning). Namely, the multiphton process is dominant here.

In Fig. 4b, the corresponding laser fields *E*(*t*′) (blue line) along with scheme of electron dynamics are presented. Note that the red lines indicate the instantaneously combined atom-field potential and the green lines are the corresponding semiclassical long trajectories. The insets labeled by **c** indicate the return energies associated with the 1^st^ returning, the 2^nd^ returning, and the 3^rd^ returning, respectively. The color map of the return energy plane indicates the dominant contributions to the trajectories. For the first return, the electron first tunnels through the lower part of the barrier potential, accelerates and returns to the core. This is a typical tunneling process at −0.8 optical cycle, when the electron once again returns to the core at −0.2 optical cycle, it now faces the combined atom-field potential wall with the higher part of the potential barrier and the tunneling is unlikely. As the discussion in Fig. 4a, this process only involves the electrons scattered off the higher part of the combined atom-field potential followed by the absorption of many photons and quickly returns to the core (the 2^nd^ returning), and the multiphton process is dominant. At the same time, electrons may have the tunneling process along the lower part of the potential barrier at −0.2 optical cycle (first return, not shown in Fig. 4b). On the other hand, for the second rescattering, the electron moves against the force field near the peak of laser field, so the electron is pulled to quickly return to the core. Next, the electron faces the lower potential barrier and enters the tunneling regime again. The third retuning regime is similar to the first return, and the fourth retuning regime is similar to the second return. Combining with the results of the insets, we find that the short trajectories with odd number times of rescattering are dominanted by the tunneling ionization mechanism at each optical cycle. On the other hand, the long trajectories with even number times of rescattering are dominanted by the multiphoton ionization mechanism.

For the dipole moment of the laser-driven atom consists of multiple rescattering events, and that each event has its own frequency characteristics, we can extend the empirical mode decomposition (EMD) technique^21^ to separate these components. The EMD technique renders a series of functions called the intrinsic mode function (IMF), on which the synchrosqueezing transform (SST)^21^,^22^ can be applied subsequently to illustrate the instantaneous frequency, via an algorithm called the sifting process^33^,^34^ as shown in the parts of method. According to the analysis in Ref. 34, if the low-to-high frequency ratio between the two neighboring IMFs is smaller than 0.67, the EMD can differentiate the two components with little coupling artifact. In this study, the ratio of multiple return component and the first return component for the *λ* = 1600 nm are approximately 0.5, implying that the first and multiple returns can be discerned by the EMD method. The time-frequency representations of the dipole moments and the IMFs are provided by SST.

**Figure 5:**
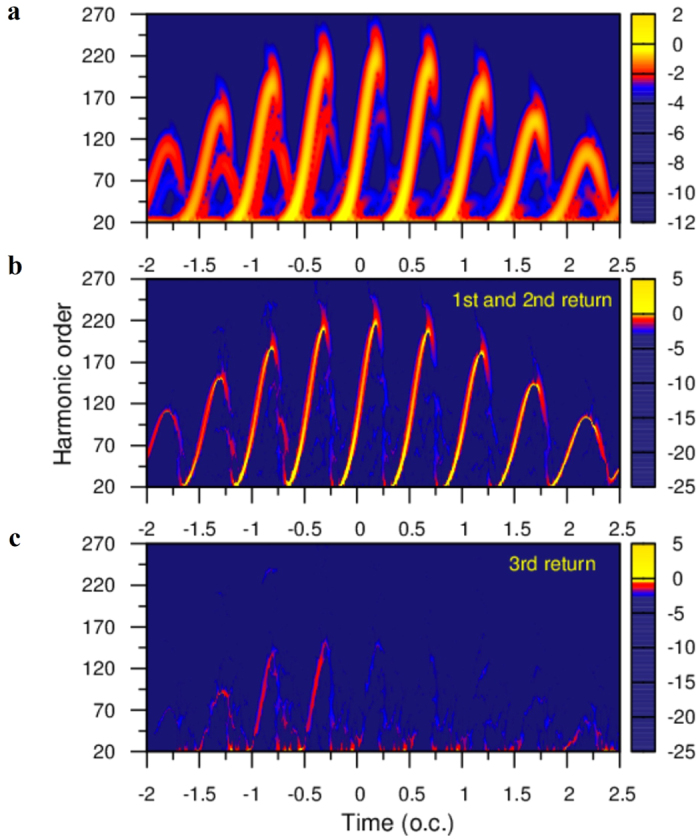
HHG time-frequency spectra and quantitative extraction of multiple rescattering from SST time-frequency spectra. (**a**) Time-frequency analysis of the HHG spectra of hydrogen atom by using STFT transform. The color bar is in a logarithmic scale. (**b**) The quantitative extraction of the 1^st^ and 2^nd^ returning contributions from SST time-frequency spectra based on EMD method coincides with the laser-driven electron first return and second return. (**c**) The quantitative extraction of the 3^rd^ returning contributions from SST time-frequency spectra. The laser parameters used are the same as those in Fig. 2a.

In Fig. 5a, the short-time Fourier transform (STFT) time-frequency representation of the dipole moment before applying the EMD method is presented. The laser parameters used are the same as those in Fig. 2a. It is readily observed that the main contribution to the harmonic generation is due to the short trajectories belongs to the first return. To show the individual multiple scattering contributions, Fig. 5b,c present the time-frequency representation of the first IMF and the second IMF, respectively. In Fig. 5b, it is evident that the short trajectories are dominant for the 1^st^ returning electrons, while the long trajectories are dominant for the second returning, particularly the contribution of the long trajectories is strong in the optical cycles before the pulse peak. In Fig. 5c, the second IMF coincide with the laser-driven electronic 3^rd^ returning to the parent ion, and the short trajectories make the most contribution before the pulse peak. Therefore, the individual multiple scattering contributions from an *ab initio* calculations are in good agreement with the extended semiclassical simulation shown in Fig. 4. As the discussion above, we can conclude that the tunneling ionization regime is responsible for the odd number times of rescattering events and the corresponding short trajectories are dominant at each half optical cycle. On the other hand, the multiphoton ionization regime is responsible for the even number times of rescattering events and the corresponding long trajectories are dominant at each half optical cycle.

## Control of Multiple Rescattering Processes for the Optimal Generation of Supercontinuum Harmonic Generation

**Figure 6:**
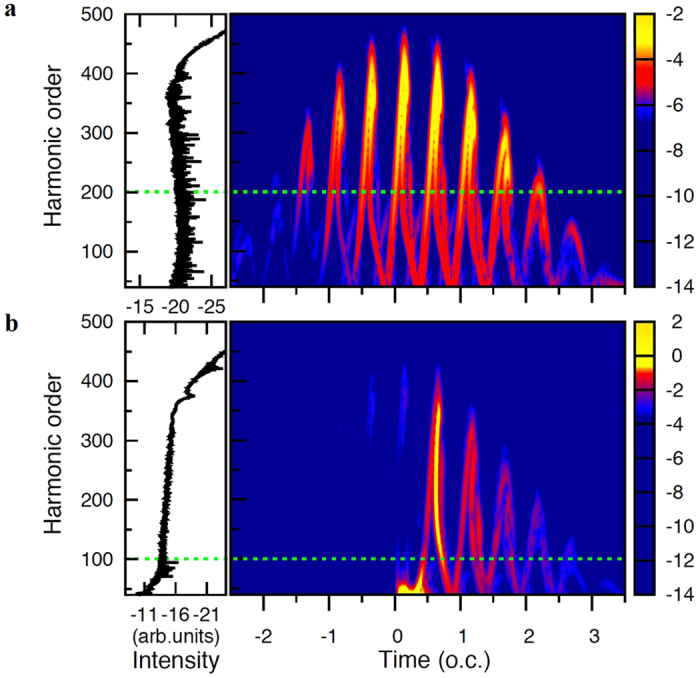
Control of multiple rescattering processes for the optimal generation of supercontinuum harmonic spectra. (**a**) The HHG (left pattern) and time-frequency analysis (right pattern) of He atom by using STFT transform in a 2000-nm mid-infrared laser field with the peak intensity*I* = 2.0 × 10^14^ W/cm^2^. The other laser parameters used are the same as those in Fig. 2a. The color bar is in a logarithmic scale. The green doted lines indicate the maximum harmonic order of the strong contributions of the multiple rescattering. (**b**) The optimal HHG and time-frequency analysis of He atom by controlling the contributions of the multiple rescattering processes.

From the discussion above, we find that the generation of the harmonic plateau includes both the contributions of the first return and multiple return. Usually the plateau itself is not the broadband supercontinuum spectra due to the sophisticated effects of multiple returns on the harmonic generation. To obtain the ultrabroad band supercontinuum spectra for the generation of the single attosecond pulse, we need to control the contributions of multiple returns in HHG. Therefore, a deeper understanding of the underlying dynamics of the multiple rescattering processes in HHG is significant and can provide fresh new insight to the control of the multiple rescattering processes to facilitate the optimal generation of the ultrabroad band supercontinuum spectra. Figure 6 shows an example of the control of the multiple rescattering processes in HHG of He atom for the ultrabroad band supercontinuum harmonic generation. In Fig. 6a, we show the HHG of He atom and the corresponding time-frequency analysis by using STFT transform in a 2000-nm mid-infrared laser field with the peak intensity *I* = 2.0 × 10^14^ W/cm^2^. The other laser parameters used are the same as those in Fig. 2a. The color bar is in a logarithmic scale. In Fig. 6b, we show the HHG of He atom and the corresponding time-frequency analysis by adding an extra VUV controlling pulse at time delay *t*_*d*_ = −0.05 optical cycle. In this calculation, a five-cycle VUV field with the intensity *I* = 1.0 × 10^12 ^W/cm^2^ is applied at time delay *t*_*d*_ = −0.05 optical cycle. The frequency of the VUV field is just equal to the energy between the ground and the lowest excited state of He atoms.

We find that the contributions of the first rescattering processes after 1.0 optical cycle are suppressed when an extra VUV controlling pulse is applied at time delay *t*_*d*_ = −0.05 optical cycle. In addition, the maximum harmonic order of the strong contributions of the multiple rescattering is located at the 200^th^ harmonic order (see green doted lines) in a 2000-nm mid-infrared laser field, while the maximum harmonic order of the strong contributions of the multiple rescattering is located at the 100^th^ harmonic order (see green doted lines) in the combined of the 2000-nm mid-infrared laser field and an extra VUV controlling pulse. A decrease of the maximum multiple rescattering location is useful to the extension of the supercontinuum harmonic spectra from the first return. In other words, the emission of all the harmonics have similar coherent properties when only the first return is chosen in the plateau of the HHG, and in principle can be superimposed with each other to produce a stronger radiation emission. Therefore, we find that the broadband supercontinuum harmonic spectra are produced near the peak of the laser field as shown in Fig. 6b. This supercontinuum harmonic generation supports an isolated 45 as pulse with a bandwidth of 124 eV by superposing the HHG from the 160^th^ to the 360^th^ order harmonics. According to the previous study^12^, the HHG can be extended to the keV energies in an ultra-strong mid-infrared laser field, while the HHG with the keV energies can be optimized by controlling of the multiple rescattering^16^.

In conclusion, we have presented an *ab initio* study of the dynamical origin of multiple rescattering processes in HHG of atoms which associate with the odd and even number times of returning of the electron to the parent ion. The use of EMD technique along with SST allows the unambiguous classification of individual contributions in multiple rescattering processes for the first time. By comparing the decomposition of the SST time-frequency spectra and the extended semiclassical calculations, we uncover that the short trajectories are dominant in odd number of rescattering events in each half optical cycle, while the long trajectories are dominant in the even number of rescattering events at each half optical cycle. In addition, tunnel ionized electrons mainly generate harmonics at odd number of returns and multiphoton ionized electrons mainly generate harmonics at even number of returns. Our present study and the results provide fresh new insight regarding the control of the multiple rescattering processes for the optimal generation of ultrabroad-band supercontinuum harmonic spectra and the production of single ultrashort attosecond laser pulse.

## Methods

### Bohmian trajectories

For atoms in linearly polarized laser fields, the angular momentum projection onto the polarization direction of the field (the *z*-axis) is conserved. That means the dependence of the wave function on the angle *φ* (rotation angle about the *z*-axis) is reduced to the factor exp(*imφ*), where *m* is the angular momentum projection. For *m = 0* the wave function does not depend on *φ* at all, thus the gradient of the wave function *ψ* can be calculated with respect to the coordinates *r* (radial coordinate) and *θ* (angle between the radius-vector and *z*-axis):


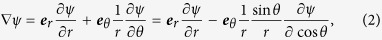


***e***_*r*_ and ***e***_*θ*_ are the unit vectors of spherical coordinate system. The equation for the Bohmian trajectories^35^ reads as


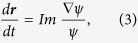


and the Bohmian trajectory lies in the plane defined by the initial (at *t* = *t*_0_) radius-vector and the *z*-axis.

### Time-dependent Schrödinger equation

A theoretical description of the HHG involves to solve the TDSE in intense laser fields. In our calculation, the TDSE is solved in space and time by means of the time-dependent generalized pseudospectral method (TDGPS)^19^. Once the time-dependent wave function is obtained, we can calculate the expectation value of the induced dipole moment, and the HHG power spectra can be calculated by the Fourier transformation of time-dependent dipole moment.

### Time-frequency transform

Here we consider the short-time Fourier transform (STFT)-based SST^22^, and choose the Gaussian function with a standard deviation of *σ* > 0 as the window function, i.e., 
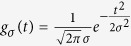
. The STFT with respect to the window *g*_*σ*_ is defined by


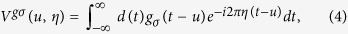


where *u* ∈ *R* is the time and *η* ∈ *R*^+^ is the frequency. The SST is given as


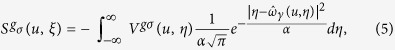


where *α* > 0 is a controllable smoothing parameter for the resolution, which in practice is chosen to be small, and 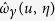 is the reallocation rule with a threshold *γ* ≥ 0: 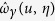 is the reallocation rule, it is written as


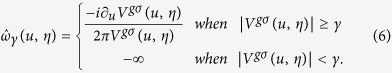


Based on a chosen linear-type transform, for example, STFT SST as a special case of reassignment methods, manifests sharpened oscillatory characteristics according to the reallocation rule. Note that since only the frequency reassignment operator is considered in SST, the causality property of the dipole moment is preserved, leading to an inverse transform.

### Empirical mode decomposition method

In this study, we perform the quantitative extraction of the multiple rescattering contributions from SST time-frequency spectra bashed on EMD method^21^. The EMD decomposes the dipole moment *d*(*t*) into a series of functions called intrinsic mode functions (IMF) via an algorithm called the sifting process. An IMF satisfies the narrow band condition, meaning that in the whole data set of a signal, the number of extrema and the number of zero crossings must either be equal or differ at most by one. In addition, at any point of an IMF, the mean value of the envelope defined by the local maxima and the envelope defined by the local minima is zero. The EMD described as follows: (i) Identify all local maxima and minima of *d*(*t*). (ii) Use a cubic spline to interpolate between the minima, resulting in an envelope curve *e*_min_(*t*). Repeat for the process for the maxima and obtain *e*_min_(*t*). (iii) Compute the mean of the envelope curves: 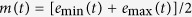. (iv) Subtract the mean from the signal and obtain *s*_1_(*t*): *s*_1_(*t*) = *d*(*t*) − *m*(*t*). Repeat step (i) to (iv) on *s*_1_(*t*) to obtain *s*_2_(*t*).We denote *s*_*k*_(*t*)as the result of the *k*th iteration.

For each iteration, we perform time-frequency analysis on *s*_*k*_(*t*).When the second return components on the time-frequency representation is removed, say on the *n*th iteration, the sifting process is stopped, and *IMF*_1_(*t*) = *s*_*n*_(*t*). Note that *IMF*_1_(*t*) not only comprises the first return components, but also the third return as well as other multiple returns. Although further iteration can provide a clean extraction of the first return components, the interpolation process in step (ii) may introduce artificial information and lead to inaccurate physical description.

## Additional Information

**How to cite this article**: Li, P.-C. *et al*. Exploration of laser-driven electron-multirescattering dynamics in high-order harmonic generation. *Sci. Rep.* **6**, 32763; doi: 10.1038/srep32763 (2016).
